# Unlocking the Potential of the Antimicrobial Peptide Gomesin: From Discovery and Structure–Activity Relationships to Therapeutic Applications

**DOI:** 10.3390/ijms24065893

**Published:** 2023-03-20

**Authors:** Xiaorong Liu, Sónia T. Henriques, David J. Craik, Lai Yue Chan

**Affiliations:** 1 Institute for Molecular Bioscience, Australian Research Council Centre of Excellence for Innovations in Peptide and Protein Science, The University of Queensland, Brisbane, QLD 4072, Australia; 2 Translational Research Institute, Faculty of Health, School of Biomedical Sciences, Queensland University of Technology, Brisbane, QLD 4102, Australia

**Keywords:** antimicrobial, anticancer, gomesin, β-hairpin, disulfide bonds, drug design

## Abstract

Gomesin is a cationic antimicrobial peptide which is isolated from the haemocytes of the Brazilian tarantula Acanthoscurria gomesiana and can be produced chemically by Fmoc solid-phase peptide synthesis. Gomesin exhibits a range of biological activities, as demonstrated by its toxicity against therapeutically relevant pathogens such as Gram-positive or Gram-negative bacteria, fungi, cancer cells, and parasites. In recent years, a cyclic version of gomesin has been used for drug design and development as it is more stable than native gomesin in human serum and can penetrate and enter cancer cells. It can therefore interact with intracellular targets and has the potential to be developed as a drug lead for to treat cancer, infectious diseases, and other human diseases. This review provides a perspective on the discovery, structure–activity relationships, mechanism of action, biological activity, and potential clinical applications of gomesin.

## 1. Introduction

Multidrug-resistant bacteria, also known as superbugs, have become a serious hazard to public health, and antimicrobial resistance has been declared to be one of the top ten global public health threats facing humanity. The social and economic impact of antimicrobial resistance is huge because of prolonged hospital stays and the need for expensive medications [1,2]. For example, there are more than 2.8 million antibiotic-resistant infections and more than 35,000 deaths each year in the United States [3]. As a result of resistance, traditional antibiotics are becoming increasingly ineffective, necessitating the development of better antimicrobial alternatives. In addition to existing treatment strategies, such as using conventional antibiotics used to target protein synthesis [4], antimicrobial peptides (AMPs) are thought to be a feasible alternative for eliminating multidrug-resistant bacteria because their mode of action differs from that of currently available antibiotics; they disturb cell membrane integrity [5,6], impede protein [7,8,9,10,11] and cell wall formation [12,13,14,15], and alter enzyme function [10,16,17]. AMPs can be used for simultaneous or subsequent treatments in many bacterial illnesses as they have independent therapeutic efficacies and fewer adverse effects than conventional antibiotics [18].

**Figure 1:**
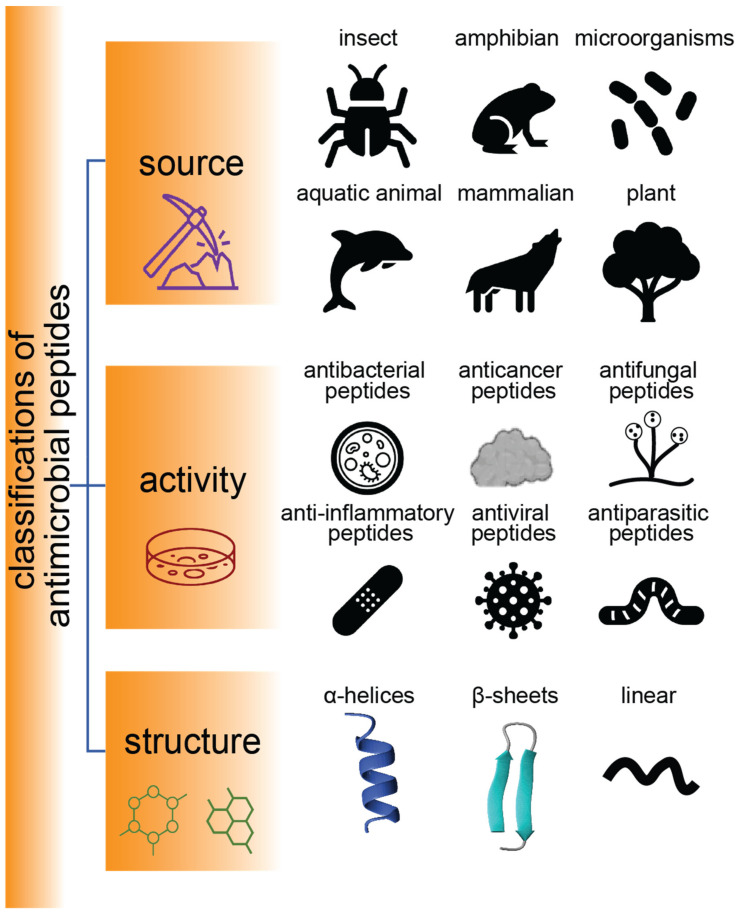
Classification of AMPs. Schematic representation of sources, activities, and structures of AMPs.

AMPs are found throughout nature and play a vital role in the innate immune systems of many species. They have a wide spectrum of biological activities (e.g., antimicrobial, antifungal, antiparasitic, and antiviral properties) and a wide range of secondary structures, as highlighted in Figure 1 [19]. With rising numbers of drug-resistant bacteria and an increasing awareness of the misuse of antibiotics, AMPs are being considered as alternative antimicrobial therapies and are a major focus of research across the world, with promising applications in health, food, animal husbandry, agriculture, and aquaculture. Mammals, amphibians, microbes, and insects are the main source of AMPs, according to statistics reported by Duwadi et al. [20]. AMPs discovered in aquatic animals (e.g., tachyplesin from *Tachypleus tridentatus* and magainin from *Xenopus laevis*) and terrestrial animals (e.g., androctonin from the scorpion *Androctonus australis*, and protegrins from porcine leukocytes) have also received much interest [21].

Several classes of insect AMPs have been reviewed recently [22]. The current report focuses on a β-hairpin peptide known as gomesin (Gm), which is still relatively understudied. This 18-amino-acid antimicrobial peptide is isolated from the haemolymph of the tarantula spider, *Acanthoscurria gomesiana,* and inhibits bacterial growth as well as the development of filamentous fungus and yeast [23]. Apart from its antimicrobial activity, its cytotoxicity to cancer cells gives Gm anticancer properties. Given some of the drawbacks of current anticancer chemotherapy, including substantial side effects and/or the development of drug resistance, there is interest in exploring gomesin as an anticancer drug. To date, there are many anticancer drugs on the market, but only 10% are in the peptide category [24,25]. Since peptides are generally considered a safer alternative than traditional cytotoxic drugs, they have recently gained interest as potential anticancer therapeutics.

**Figure 2:**
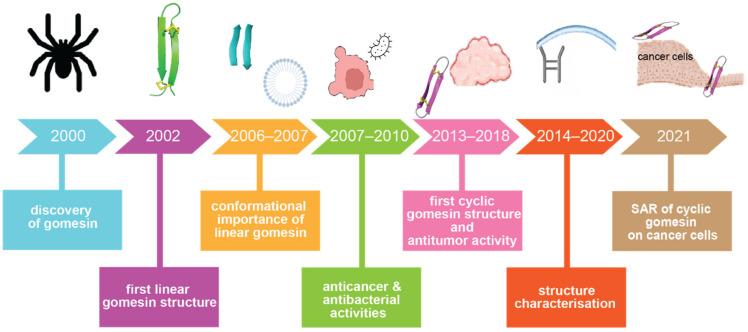
Historical timeline of gomesin studies. Key milestones in understanding the structure and activities of gomesin are indicated. Abbreviation: SAR: structure–activity relationship.

Since the discovery of Gm in 2000 [23], it has undergone more than 20 years of research, as summarised in Figure 2. To begin to understand its functions, the first three-dimensional solution structure of gomesin (PDB ID: 1KFP) was reported in 2002 [26], which was followed by a structure–activity relationship study in 2006 [27]. From 2007 to 2018, a range of bioactivities of Gm were characterised (including anticancer and antimalarial activities), and the chemical synthesis of a series of analogues with various chemical modifications were reported, including those involving amino acid mutations [28], with/without disulfide bonds [29], and backbone cyclization [30]. The first backbone cyclization study on gomesin was reported in 2013 and included a three-dimensional structure of the cyclic form [31]. Several structure–activity studies based on cyclic gomesin were reported thereafter, with a focus on optimizing antimicrobial and anticancer properties of cyclic gomesin analogues.

## 2. Discovery, Synthesis and Structural Characterization of Gomesin

**Figure 3:**
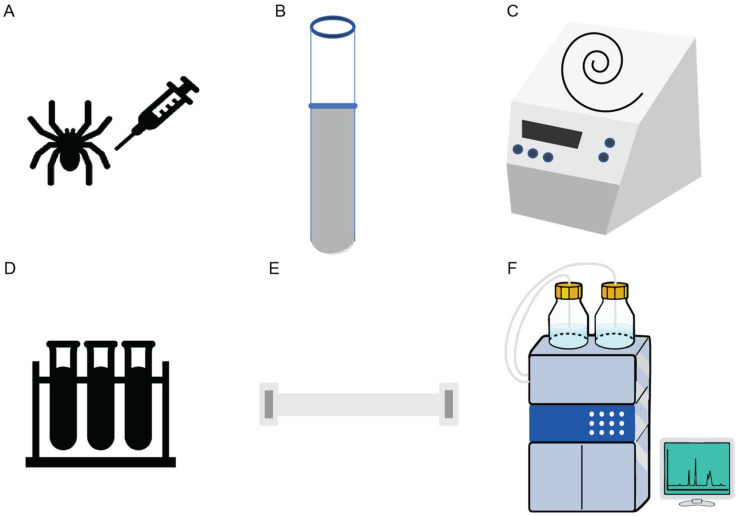
Schematic illustration of the isolation of Gm from spiders, hemolymph collection, and peptide extraction and purification. (**A**) Hemolymph collection from a spider is typically performed in sodium citrate buffer (pH 4.6). (**B**) Hemocytes are collected from the hemolymph. (**C**) Concentration and pyrolysis steps are done using vacuum centrifugation. (**D**) Solid phase extraction using pre-purified supernatant from ultrasonic treatment is then applied. (**E**) Aquapore RP-300 C_8_ column for peptide elution. (**F**) Further purification of the active peptide through high performance liquid chromatography-size exclusion column (HPLC-SEC_1_) completes the process [23].

Figure 3 schematically illustrates the steps involved in the isolation and structural characterization of gomesin. Through initial peptide sequencing and structural characterization, gomesin was reported to comprise 18 amino acids, including a pyroglutamic acid residue at the N-terminus, an amidated arginine at the C-terminus, and 4 cysteine residues that form 2 disulfide linkages (ZCRRLCYKQRCVTYCRGR-NH_2_), and with a molecular weight of 2270.4 Da [23]. In that first study Edman degradation was performed to determine the peptide sequence, but more recent studies of peptides typically use MS based peptide sequencing.

While many early studies of gomesin involved the use of natively isolated peptide, the development of synthesis methods to make gomesin was important for establishing structure–activity relationships. In general, currently marketed therapeutic peptides, or those made for laboratory studies, are typically produced by either chemical synthesis or recombinant DNA technology [32]. So far solid-phase peptide synthesis (SPPS) is the most used method to produce Gm [27]. Using Fmoc (fluorenyl methoxycarbonyl) based protection chemistry, peptides are typically assembled from the carboxyl terminus (an amidated C-terminus in the case of gomesin) to the amino terminus (N-terminus with a pyro-glutamic acid residue in the case of gomesin) of the amino acid chain. By contrast, in a natural cell environment, peptides are synthesised from the N- to the C-terminus [33].

**Figure 4:**
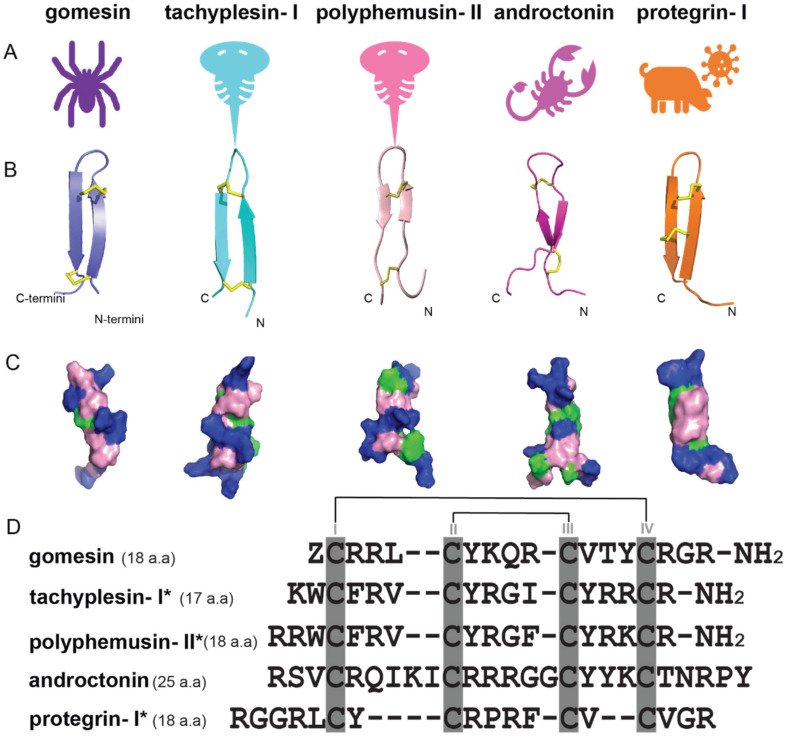
Structures and sequence alignment of β-hairpin AMPs. (**A**) Natural sources from which listed peptides were first identified: gomesin was isolated from the spider *Acanthoscurria gomesiana*; tachyplesin-I from the horseshoe crab *Tachypleus tridentatus*; polyphemusin-II from the horseshoe crab *Limulus polyphemus*; androctonin from the scorpion *Androctonus australis*; protegrin-I from the porcine leukocytes (*Sus domesticus*). (**B**) Ribbon representation of the β-hairpin-like structure of Gm (PDB ID: 1KFP), tachyplesin (PDB ID: 2RTV), polyphemusin (PDB ID: 1RKK), androctonin (PDB ID: 1CZ6), and protegrin (PDB ID: 1PG1). Disulfide bonds are represented as yellow sticks. (**C**) Surface representations of Gm, tachyplesin-Ⅰ, polyphemusin-II, androctonin, and protegrin-Ⅰ. Non-polar (hydrophobic), polar, and positively charged residues are shown in green, pink, and blue, respectively. (**D**) Sequence alignment and total amino acid (a.a) content of Gm, tachyplesin-Ⅰ, polyphemusin-II, androctonin, and protegrin-Ⅰ. Cysteine residues are highlighted by a grey box, and disulfide bond connectivities (Cys I-IV, and Cys II-III) are shown in thick black lines. *: There is more than one variant of this peptide [35,36,37,38,39,40].

The amidated C-terminal arginine residue of gomesin has been reported to be important for minimizing enzymatic degradation, improving peptide stability, and with the potential to enhance antimicrobial activity due to the protonation of the sidechain of this C-terminal residue [20,34]. It is thus of interest to explore the prevalence of this charged C-terminal motif in other AMPs. Figure 4 shows a comparison of Gm with antimicrobial peptides from other arthropods (e.g., tachyplesin-Ⅰ [35,36], polyphemusin-II [37], androctonin [38]) and porcine leukocyte families (e.g., protegrin-Ⅰ [39]) and reveals high sequence and structural similarity. These peptides all comprise antiparallel β-sheets which contribute to their high stability profiles [40]. It is noteworthy that the N- and C-termini of these peptides are close to each other, leading to the possibility that they could be joined chemically to produce cyclic derivatives, as described later in this article.

**Figure 5:**
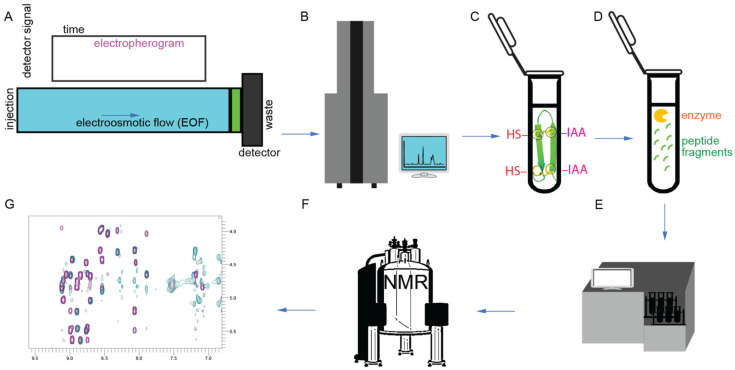
Structural characterization workflow. (**A**) Capillary zone electrophoresis. (**B**) MALDI-TOF-MS and electrospray ionization mass spectrometry. (**C**) Reduction and alkylation steps. -HS (thiol groups; dithiothreitol was used to reduce disulfide bonds) and -IAA (iodoacetamide was used for alkylation). (**D**) Trypsin digestion step. (**E**) Amino acid sequencing. (**F**) NMR spectrometer. (**G**) NMR two-dimensional (2D) spectrum [23,26].

The procedures for determining the structures of gomesin [23,26] and related peptides are shown in Figure 5. For gomesin, capillary zone electrophoresis was used to separate peptides in a haemolymph extract, and masses were confirmed by MALDI-TOF mass spectrometry (Figure 5A,B). Enzyme digestion and alkylation steps were performed on purified peptides followed by amino acid sequencing for sequence confirmation (Figure 5C–E). Two-dimensional (2D) proton NMR (Figure 5F,G) was used to confirm the peptide sequence and 3D overall structure. It was found that the global fold of gomesin is made up of two antiparallel β-strands joined by a four-residue non-canonical turn (Figure 5B). The β-strands are connected by two interchain disulfide bonds, and six interchain skeleton-framework hydrogen bonds further stabilise the overall structure [41]. Both disulfide bonds adopt a right-handed conformation with a twist angle close to the low-energy conformation found in disulfide bonds bridging antiparallel β-strands, which is similar to other antimicrobial peptides [27].

Detailed analyses of the disulfide bond geometries, β-hairpin geometries, charges, and bioactivities for a selection of β-hairpin peptides are given in Table 1. The Cysα-Cysα distances across the two disulfide bonds in gomesin are 0.38 ± 0.10 nm (Cys6-Cys11) and 0.37 ± 0.10 nm (Cys2-Cys15). These distances are much shorter than typical Cα-Cα distances connecting other secondary structure motifs or β-sheet disulfide bonds, which is more than 0.45 nm, as found in larger, more spherical peptides/proteins. This short Cysα-Cysα distance is thus a useful marker for high stability.

**Figure 6:**
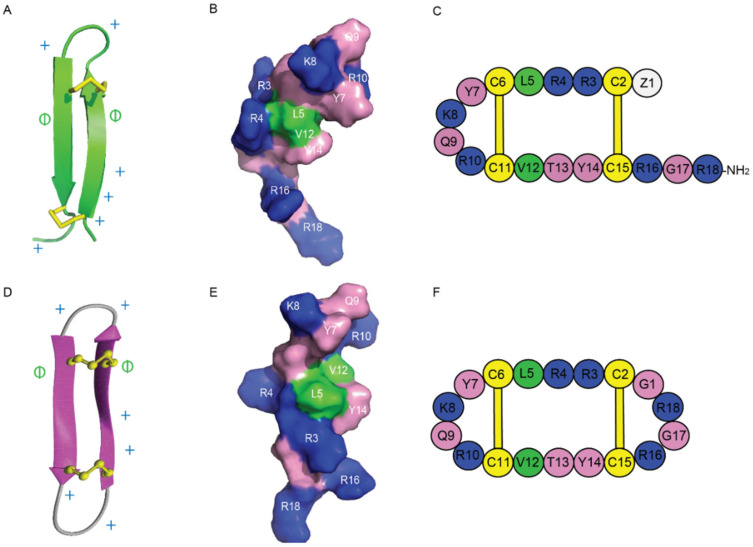
Structures of Gm and cGm. (**A**) Ribbon representation of the β-hairpin structure of Gm with disulfide bonds highlighted in yellow. PDB structure ID: 1KFP. (**B**) Surface representation of Gm. Non-polar (hydrophobic) residues are shown in green, polar residues in pink, and positively charged residues in blue. (**C**) A schematic view of Gm sequence. (**D**) Three-dimensional structure of cGm. Disulfide bonds are represented with yellow ball and sticks, and two antiparallel β-sheets are shown in purple. BMRB ID: 17986. (**E**) Surface representation of cGm. Non-polar (hydrophobic) residues are shown in green, polar residues in pink, and positively charged residues in blue. (**F**) A schematic view of cGm sequence. Positively charged residues are indicated with a ‘+’ symbol, hydrophobic residues are indicated with a ‘ɸ’ symbol on (**A**,**D**).

The amphiphilic nature of Gm is another feature commonly shared by other AMP/anticancer peptides. The stable β-hairpin-like structure of Gm, as well as its positively charged and amphiphilic properties, make it highly potent with selectivity to certain cancer cell types, such as melanoma or chronic myeloid leukaemia cells [42]. Based on these findings and the proximity of the termini in gomesin, cyclic gomesin (cGm) has been used as a scaffold for drug design. It comprises a cyclic version of the native Gm with a backbone joined at the N- and C- terminus via the addition of a glycine linker residue [31]. Its structure, shown in Figure 6, has been determined and the cyclic peptide has been shown to be more stable than its linear counterpart. It enters cancer cells via a mechanism modulated by electrostatic interactions between its positively charged surface exposed residues [42,43], illustrated in Figure 6, and the negatively charged phospholipids at the outer layer of cancer cell membranes.

## 3. Chemical Methods to Derive Structure–Activity Relationships

**Figure 7:**
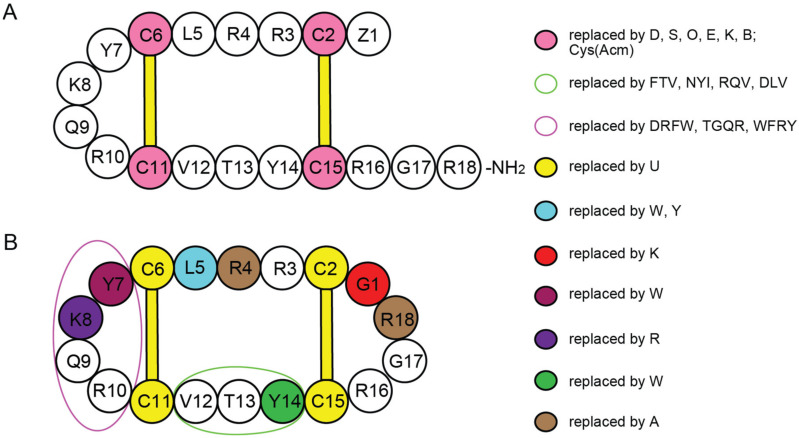
Residue modifications of Gm and cGm analogues. (**A**) Gm sequence with modified residues. (**B**) cGm sequence with modified residues. The coloured circles represent modified residues. The two disulfide bonds in each peptide are shown (yellow). Three residues replacement are highlighted with a green circle and four residues replacement are highlighted with a purple circle. A, alanine; B, diaminopropionic acid; C, cysteine; D, aspartic acid; E, glutamic acid; F, phenylalanine; G, glycine; I, isoleucine; K, lysine; L, leucine; N, asparagine; O, ornithine; Q, glutamine; R, arginine; S, serine; T, threonine; U, selenocysteine; V, valine; W, tryptophan; Y, tyrosine.

Establishing SAR within a peptide family is a basis for designing peptides with higher potency and selectivity against bacteria or cancer cells than native peptides alone [41]. The majority of Gm SAR investigations have focused on the role of disulfide bonds on biological activity and conformation [27], as well as the influence of hydrophobicity or charge alterations on membrane binding, cleavage, or cytotoxic action [41]. Approaches that have been applied include substituting cysteine residues with serine or tyrosine, adding Acm protecting groups on cysteine residues, creating lactam mono or bicyclic analogues [27], and the use of cysteine-free analogues. Some of these approaches improved the antimicrobial activity, and others confirmed that the disulfide bridges are required to maintain β-hairpin structure and bioactivity of Gm [44]. Figure 7 summarises amino acid modifications to cGm that have been reported and are more stable than native Gm [23,27,31,42,45,46,47,48,49,50].

## 4. Biophysical Studies to Elucidate the Mechanism of Action Gomesin on Bacterial and Mammalian Cell Membranes

### 4.1. Membrane Binding and Peptide–Lipid Interactions

Most AMPs, including Gm, exert their cytotoxic effects via membrane permeabilization. Since membrane binding is required for permeabilization, researchers have investigated the binding of Gm and its variants to cell membranes using a range of techniques [41]. The cytoplasmic membrane is widely established to be a target of cationic AMPs. Typically, such cationic AMPs interact with negatively charged phospholipid head groups and enter the cell via direct membrane permeation [32]. Thus, like most other AMPs, Gm has a higher binding affinity and specificity for negatively charged bacterial membrane surfaces [31].

### 4.2. Permeabilization of Cell Membranes and Leakage Activity

Gm permeabilises cell membranes, as has been demonstrated with membrane leakage experiments using lipid vesicles, bacteria, yeast, and cancer cells [42]. Gm seems to permeabilise membranes via a lipid-dependent process without the participation of cell surface receptors or other membrane proteins found in the plasma membrane [51,52]. This membrane permeabilization has also been used to investigate Gm activity and selectivity on certain cancer and/or bacterial cells [53]. Gm induced LDH release in a concentration-dependent manner on murine melanoma cells (B16F10) [45], human neuroblastoma cells (SH-SY5Y), and rat pheochromocytoma cells (PC12) [54], confirming disruption of cell membranes.

## 5. Modifications of Gm and cGm

The design of new Gm and cGm analogues is an emerging topic in the field of β-hairpin peptides. In an early study, Fazio et al. [27] were interested in the structure-activity relationships of Gm and noted that monocyclic disulfide-bridged analogues exhibited different antimicrobial activities. More recently, Henriques et al. [42] focused on cGm-modified analogues and reported several that had higher toxicity against melanoma and leukaemia cells than cGm itself. Most recently, Nadal-Bufi et al. used cGm as a scaffold to design grafted analogues [49]; the grafted peptides potently inhibited the enzymatic activity of LDH, a biomarker for many cancers that is involved in altered metabolism and tumour proliferation. Some recent examples from the studies of Henriques et al. [42] and Nadal-Bufi et al. [49] with applications to anticancer and antimicrobial activities are summarised in Table 2.

## 6. Biological Activity of Gomesin and Analogues

### 6.1. Antimicrobial Activity

Gm, cGm, and analogues exhibit strong activity against almost all Gram-negative bacteria tested (e.g., *Escherichia coli*, *Pseudomonas aeruginosa*, *Salmonella*, and *Klebsiella pneumoniae*) and some Gram-positive, including *Staphylococcus aureus* [23,27,31,42,47] in planktonic state. Recently, the [G1K,R8K] cGm analogue was shown to also display against biofilms of *Staphylococcus aureus* [50]. The antimicrobial potency of Gm and analogues is thought to be modulated by several structural properties, including: (i) double-stranded antiparallel-sheet structure; (ii) disulfide connectivity; and (iii) amphipathic properties and overall charge. Alterations in the peptide sequence modifying these properties typically changes the antimicrobial potency [27,42,50,55].

### 6.2. Anticancer Activity

Data from a range of tested cancer cell lines suggest that Gm has anticancer activity [42], but displays selectivity for certain types of cancers, as it is more effective against melanoma cell lines (e.g., MM96L; the concentration required to induce cytotoxicity to 50% of cells (CC_50_) is 5.5 ± 1.1 μM) and against chronic myeloid leukaemia cells (i.e., K562; CC_50_ is 5.5 ± 1.1 μM) compared to other cancer cell lines (e.g., CC_50_ of 72.7 ± 1.5 μM against cervical cancer cells HeLa). The capacity of Gm to selectively target and disrupt negatively charged cancer cell membranes appears to be linked to its anticancer activity, but other cell membrane properties seem to further improve sensitivity towards melanoma and chronic myeloid leukemia [42].

cGm is a stable peptide that targets and enter cancer cells with higher efficacy than well-characterized cell-penetrating peptides such as TAT. cGm binds to the cell membrane via electrostatic interactions between positively charged peptides and negatively charged phospholipid head groups exposed on the cell surface; it enters cells via endocytic mechanisms and direct membrane penetration [43]. The analogue, [R/r] cGm, where the fourth (R4), tenth (R10), and eighteenth (R18) arginine residues were replaced with the enantiomeric residue, d-arginine [43], retains the capacity to bind with high affinity to negatively charged lipid membranes; it enters cancer cells and has the advantage of being non-membrane disruptive and non-toxic against tested cell lines [43]. Based on these findings, [R/r] cGm would be an excellent scaffold to target and deliver therapeutic compounds into cancer cells while causing minimal harm to healthy cells.

### 6.3. Antiprotozoal Activity

In addition to antimicrobial and anticancer activities, Gm has antiprotozoal activity. Specifically, Gm has activity against epimastigote forms of *Trypanosoma cruzi*, two flagellate protozoa (i.e., *Leishmania amazonensis* and *Leishmania mayor)* and against ookinetes of plasmodium [48]. Its anti-parasitic mechanism is likely to involve permeabilization of cell membranes, as suggested in a study by Moreira et al. with *Plasmodium falciparum* and *P. berghei* [48]. Furthermore, in an in vivo mode, Gm was found to have the ability to inhibit the growth of oocysts of *Plasmodium falciparum* and *P. berghei* species in *Anopheles stephensi* mosquitoes [48]. Given the increasing resistance of plasmodium parasites to commonly used drugs and the resistance of mosquitoes to insecticides [48], Gm was proposed as a promising anti-plasmodium candidate and an excellent candidate for transmission blockers of mosquito genetic engineering [32].

The cyclic derivative, cGm, displays higher anti-parasitic potency than linear Gm, as shown by the work of Moreira et al. [48] and Chan et al. [31]. For example, Chan et al. demonstrated higher anti-parasitic activity for cGm when tested on the chloroquine-resistant W2 strain of *P. falciparum* [31]. Once again, this illustrates that the backbone cyclization of gomesin is a convenient and efficient way to improve its properties for pharmaceutical applications.

### 6.4. Therapeutic Efficacy and Safety Profile on Human Red Blood Cells

Gm has some toxicity to human red blood cells (16% haemolysis at 1 μM) [23]; however, this undesired activity can be reduced or eliminated by structural modification, while increasing the desired activity (e.g., antimicrobial activity) and thereby the therapeutic index. For instance, cGm is less haemolytic than Gm [31], which means the therapeutic index of cGm is greater than native Gm. Moreover, the disulfide bonds of Gm have been shown to play an important role in haemolytic activity, and the removal of one or two bridges reduces the haemolytic activity of both linear and monocyclic analogues [27].

## 7. Future Outlook and Conclusions

The applications of natural peptides as therapeutics have in the past been hampered by their poor pharmacokinetic features, since they have a low capacity to traverse physiological barriers and are rapidly destroyed by digestive enzymes [56,57]. Furthermore, when compared to traditional small molecule drugs, peptides are potentially more expensive to produce chemically, and may not be as stable [57,58]. However, Gm is a unique type of AMP with some useful advantages. It is naturally produced in spiders but is expressed in limited amounts. Therefore, optimizing its chemical synthesis yield is important for the future scalable production for pharmaceutical applications. Due to its relatively small size (i.e., only 18 aa residues), large-scale production may be feasible at a low cost. Overall, the benefits and multifunctional biological properties of Gm and its derivatives suggest a promising future in pharmaceutical applications.

cGm offers several benefits over Gm. For example, some analogues have been developed with greater specificity against microorganisms than human red blood cells [32], so they can exert antimicrobial effects without haemolysis. The most notable consideration is that cGm is a more stable and less toxic analogue than Gm. This enhanced analogue has better capability to enter cancer cells and can be employed as a scaffold for designing new anticancer peptide therapeutics [43].

It has been more than two decades since the initial discovery and isolation of Gm; however, it is still mainly involved in preclinical stages, indicating more in vivo model research is needed to achieve further progression as a lead for microbial infections or cancer treatments [41]. Some peptides, similar in structure to Gm, have progressed into clinical trials or are on the market as anti-infectious peptide drugs. For example, IntraBiotics Pharmaceuticals (IBPI), a US-based company, produced a synthetic version of protegrin called Iseganan (IB367, protegrin IB367), which is a new generation of antimicrobial for the prevention and treatment of major infectious illnesses. Protegrin is a small soluble β-hairpin peptide derived from swine leukocytes. IBPI reported the successful completion of a Phase II clinical investigation of Iseganan as a mouthwash for the prevention and treatment of oral mucositis. Currently, it is in Phase III clinical trial (clinical trial ID: NCT00022373) under investigation for head and neck cancer patients who received radiotherapy [59,60]. Iseganan also entered Phase I clinical trials as an aerosol for the treatment of respiratory infections in cystic fibrosis patients [32]. Based on this example, apart from its antimicrobial activity, Gm could be used to target respiratory diseases in the future since ongoing pandemic diseases, such as COVID-19, have not been fully resolved, and complications include pneumonia and severe lung inflammation. Current major preclinical studies on Gm have been heavily focused on antimicrobial, antiprotozoal, and anticancer applications. Other applications, such as respiratory diseases, wound healing and skin infections, intestine infection, and inflammation, could be further explored to maximize its success in progressing to clinical stages.

Gomesin could also be further improved for future drug delivery purposes depending on the desired route of administration and treatments needed in actual clinical settings. For example, chemical strategies such as N-methylation could be employed to enhance oral bioavailability if an oral administration route is required [61]. Other chemical strategies, such as lipidation, PEGylation, and albumin fusion, could play a role in the half-life extension of biopharmaceuticals such as gomesin [62].

In conclusion, this review has provided an in-depth analysis of gomesin in terms of its discovery, structure–activity relationships, and mechanisms of action. Future studies on this peptide are still required to further progress into clinical trials for medicinal applications.

## Figures and Tables

**Table 1:** Structural properties of antimicrobial peptides with β-hairpin-like structure.

Antimicrobial Peptides	Function/Activity	Disulfide Connectivity	Cysα-Cysα Distances (nm)	Types of β-Sheets	Total Net Charge
gomesin	Antimicrobial, anticancer	Cys2-Cys15, Cys6-Cys11	0.37 ± 0.10, 0.38 ± 0.10 (<0.45) [41]	right-handed rotamer, antiparallel β-sheets	+6
tachyplesin-I	Antimicrobial, antifungal, and anticancer	Cys3-Cys16, Cys7-Cys12	0.43 ± 0.20, 0.39 ± 0.20 [40]	right-handed rotamer, antiparallel β-sheets	+6
polyphemusin-II	Antimicrobial and antifungal	Cys4-Cys17, Cys8-Cys13	0.43 ± 0.50, 0.37 ± 0.10 [40]	right-handed rotamer, antiparallel β-sheets	+7
androctonin	Inhibits the growth of Gram-positive bacteria	Cys4-Cys20, Cys10-Cys13	n.a.	right-handed rotamer, antiparallel β-sheets	+8
protegrins-I	Antimicrobial and antifungal	Cys6-Cys15, Cys8-Cys13	0.38 ± 0.30, 0.35 ± 0.10 [40]	right-handed rotamer, antiparallel β-sheets	+6

n.a., not available; Cys: cysteine.

**Table 2:** Examples of Gm and cGm analogues with anticancer or antimicrobial activities.

Peptide	Applications	CC_50_/IC_50_/MIC *	References
gomesin 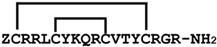	anticancer, antimicrobial, antifungal	K-562; 3.8 ± 0.3 μM *E. coli* ATCC 25922; 4 μM *C. albicans ATCC 90028;* 8–16 μM	[42]
cyclic gomesin 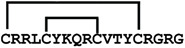	anticancer, antimicrobial, antifungal	K-562; 2.7 ± 0.1 μM *E. coli ATCC 25922*; 4 μM *C. albicans ATCC 90028;* 4–8 μM	[42,49]
[Y7W]cGm 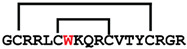	anticancer antimicrobial	K-562; 3.9 ± 0.2 μM *E. coli ATCC 25922*; 1–2 μM	[42,49]
[Y14W]cGm 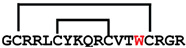	anticancer, antimicrobial	K-562; 2.7 ± 0.1 μM *E. coli ATCC 25922*; 1–2 μM	[42]
[K8R]cGm 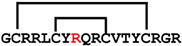	anticancer, antimicrobial	K-562; 3.1 ± 0.1 μM *E. coli ATCC 25922*; 1–2 μM	[42]
[Y7W,K8R,Y14W]cGm 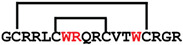	anticancer, antimicrobial	K-562; 3.9 ± 0.1 μM *E. coli ATCC 25922*; 1 μM	[42]
[R4A,R18A]cGm 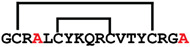	anticancer, antimicrobial, antifungal	K-562; 11.5 ± 0.6 μM *E. coli ATCC 25922*; 8 μM *C. albicans ATCC 90028;* 32 μM	[42]
[G1K,K8R]cGm 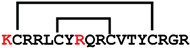	anticancer, antimicrobial, antifungal	K-562; 2.1 ± 0.2 μM *E. coli ATCC 25922*; 0.5–1 μM *C. albicans ATCC 90028;* 2 μM	[42]
[C/U]cGm 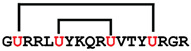	anticancer, antimicrobial, antifungal	K-562; 1.4 ± 0.2 μM *E. coli ATCC 25922*; 4–8 μM *C. albicans ATCC 90028;* 4–8 μM	[42]
[L5W]cGm 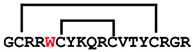	anticancer, antimicrobial	K-562; 1.0 ± 0.1 μM *E. coli ATCC 25922*; 2–4 μM	[42]
[^D^P^L^P]cGm 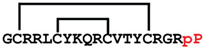	anticancer, antimicrobial, antifungal	K-562; 3.9 ± 0.2 μM *E. coli ATCC 25922*; 4 μM *C. albicans ATCC 90028;* 8 μM	[42]
[G1K,L5Y,K8R]cGm 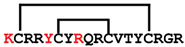	anticancer, antimicrobial, antifungal	K-562; 1.3 ± 0.1 μM *E. coli ATCC 25922*; 0.5–1 μM *C. albicans ATCC 90028;* 4 μM	[42]
[C/U,G1K,L5Y,K8R]cGm 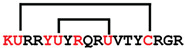	anticancer, antimicrobial, antifungal	K-562; 6.4 ± 0.6 μM *E. coli ATCC 25922*; 1–2 μM *C. albicans ATCC 90028;* 4 μM	[42]
cGmC4 ** 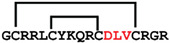 **	anticancer	LDH5 inhibitor; 27.3 μM	[49]
cGmC5 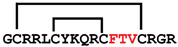	anticancer	LDH5 inhibitor; 24.0 μM	[49]
cGmN5 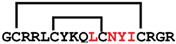	anticancer	LDH5 inhibitor; >40 μM	[49]
cGmN6 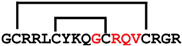	anticancer	LDH5 inhibitor; 41.4 μM	[49]
cGmC6 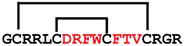	anticancer	LDH5 inhibitor; 10.4 μM	[49]
cGmC7 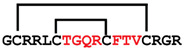	anticancer	LDH5 inhibitor; >40 μM	[49]
cGmC8 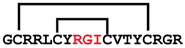	anticancer	LDH5 inhibitor; 24.8 μM	[49]
cGmC9 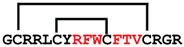	anticancer	LDH5 inhibitor; 2.5 μM	[49]
cGmC10 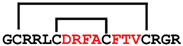	anticancer	LDH5 inhibitor; >40 μM	[49]
cGmC11 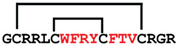	anticancer	LDH5 inhibitor; 4.5 μM	[49]

Fonts in red represent amino acid replacement; Z, pyroglutamic acid; U, selenocysteine; p, _D_-proline. * Selected examples for each application.

## Data Availability

Not applicable.

## References

[1] Antimicrobial Resistance Collaborators (2022). Global burden of bacterial antimicrobial resistance in 2019: A systematic analysis. Lancet.

[2] WHO Antimicrobial Resistance November 2021. https://www.who.int/news-room/fact-sheets/detail/antimicrobial-resistance.

[3] CDC About Antibiotic Resistance December 2021. https://www.cdc.gov/drugresistance/about.html.

[4] Khan S.N., Khan A.U. (2016). Breaking the Spell: Combating Multidrug Resistant ‘Superbugs’. Front. Microbiol..

[5] Chan D.I., Prenner E.J., Vogel H.J. (2006). Tryptophan- and arginine-rich antimicrobial peptides: Structures and mechanisms of action. Biochim. Biophys. Acta.

[6] Mattiuzzo M., Bandiera A., Gennaro R., Benincasa M., Pacor S., Antcheva N., Scocchi M. (2007). Role of the Escherichia coli SbmA in the antimicrobial activity of proline-rich peptides. Mol. Microbiol..

[7] Mardirossian M., Grzela R., Giglione C., Meinnel T., Gennaro R., Mergaert P., Scocchi M. (2014). The host antimicrobial peptide Bac71-35 binds to bacterial ribosomal proteins and inhibits protein synthesis. Chem. Biol..

[8] Le C.F., Gudimella R., Razali R., Manikam R., Sekaran S.D. (2016). Transcriptome analysis of Streptococcus pneumoniae treated with the designed antimicrobial peptides, DM3. Sci. Rep..

[9] Kragol G., Lovas S., Varadi G., Condie B.A., Hoffmann R., Otvos L. (2001). The antibacterial peptide pyrrhocoricin inhibits the ATPase actions of DnaK and prevents chaperone-assisted protein folding. Biochemistry.

[10] Le C.F., Fang C.M., Sekaran S.D. (2017). Intracellular Targeting Mechanisms by Antimicrobial Peptides. Antimicrob. Agents Chemother..

[11] Wronska A.K., Bogus M.I. (2020). Heat shock proteins (HSP 90, 70, 60, and 27) in *Galleria mellonella* (Lepidoptera) hemolymph are affected by infection with *Conidiobolus coronatus* (Entomophthorales). PLoS ONE.

[12] Nikaido H. (2003). Molecular basis of bacterial outer membrane permeability revisited. Microbiol. Mol. Biol. Rev..

[13] Cabib E. (2009). Two novel techniques for determination of polysaccharide cross-links show that Crh1p and Crh2p attach chitin to both beta(1-6)- and beta(1-3)glucan in the *Saccharomyces cerevisiae* cell wall. Eukaryot Cell.

[14] Spohn R., Daruka L., Lazar V., Martins A., Vidovics F., Grezal G., Mehi O., Kintses B., Szamel M., Jangir P.K. (2019). Integrated evolutionary analysis reveals antimicrobial peptides with limited resistance. Nat. Commun..

[15] Rautenbach M., Troskie A.M., Vosloo J.A. (2016). Antifungal peptides: To be or not to be membrane active. Biochimie.

[16] Shu G., Chen Y., Liu T., Ren S., Kong Y. (2019). Antimicrobial Peptide Cathelicidin-BF Inhibits Platelet Aggregation by Blocking Protease-Activated Receptor 4. Int. J. Peptide Res. Ther..

[17] Perlikowska R., Silva J., Alves C., Susano P., Pedrosa R. (2022). The Therapeutic Potential of Naturally Occurring Peptides in Counteracting SH-SY5Y Cells Injury. Int. J. Mol. Sci..

[18] Miethke M., Pieroni M., Weber T., Bronstrup M., Hammann P., Halby L., Arimondo P.B., Glaser P., Aigle B., Bode H.B. (2021). Towards the sustainable discovery and development of new antibiotics. Nat. Rev. Chem..

[19] Huan Y., Kong Q., Mou H., Yi H. (2020). Antimicrobial Peptides: Classification, Design, Application and Research Progress in Multiple Fields. Front. Microbiol..

[20] Duwadi D., Shrestha A., Yilma B., Kozlovski I., Sa-Eed M., Dahal N., Jukosky J. (2018). Identification and screening of potent antimicrobial peptides in arthropod genomes. Peptides.

[21] Lofgren S.E., Miletti L.C., Steindel M., Bachere E., Barracco M.A. (2008). Trypanocidal and leishmanicidal activities of different antimicrobial peptides (AMPs) isolated from aquatic animals. Exp. Parasitol..

[22] Wu Q., Patocka J., Kuca K. (2018). Insect Antimicrobial Peptides, a Mini Review. Toxins.

[23] Silva P.I., Daffre S., Bulet P. (2000). Isolation and characterization of gomesin, an 18-residue cysteine-rich defense peptide from the spider Acanthoscurria gomesiana hemocytes with sequence similarities to horseshoe crab antimicrobial peptides of the tachyplesin family. J. Biol. Chem..

[24] Sun J., Wei Q., Zhou Y., Wang J., Liu Q., Xu H. (2017). A systematic analysis of FDA-approved anticancer drugs. BMC Syst. Biol..

[25] De la torre B., Albericio F. (2020). Peptide Therapeutics 2.0. Molecules.

[26] Mandard N., Bulet P., Caille A., Daffre S., Vovelle F. (2002). The solution structure of gomesin, an antimicrobial cysteine-rich peptide from the spider. Eur. J. Biochem..

[27] Fazio M.A., Oliveira V.X., Bulet P., Miranda M.T., Daffre S., Miranda A. (2006). Structure-activity relationship studies of gomesin: Importance of the disulfide bridges for conformation, bioactivities, and serum stability. Biopolymers.

[28] Chalker J.M., Davis B.G. (2010). Chemical mutagenesis: Selective post-expression interconversion of protein amino acid residues. Curr. Opin. Chem. Biol..

[29] Chalker J.M., Bernardes G.J., Lin Y.A., Davis B.G. (2009). Chemical modification of proteins at cysteine: Opportunities in chemistry and biology. Chem. Asian J..

[30] Malins L.R. (2018). Peptide modification and cyclization via transition-metal catalysis. Curr. Opin. Chem. Biol..

[31] Chan L.Y., Zhang V.M., Huang Y.H., Waters N.C., Bansal P.S., Craik D.J., Daly N.L. (2013). Cyclization of the antimicrobial peptide gomesin with native chemical ligation: Influences on stability and bioactivity. ChemBioChem.

[32] Miranda A., Jouvensal L., Vovelle F., Bulet P., Daffre S., de Lima M.E., de Castro Pimenta A.M., Martin-Eauclaire M.F., Zingali R.B., Rochat H. (2009). A powerful antimicrobial peptide isolated from the Brazilian tarantula spider Acanthoscurria gomesiana. Animal Toxins: State of the Art. Perspectives in Halth and Biotechnology.

[33] Chan W., White P. (1999). Fmoc Solid Phase Peptide Synthesis: A Practical Approach.

[34] Machado A., Fazio M.A., Miranda A., Daffre S., Machini M.T. (2012). Synthesis and properties of cyclic gomesin and analogues. J. Pept. Sci..

[35] Kawano K., Yoneya T., Miyata T., Yoshikawa K., Tokunaga F., Terada Y., Iwanaga S. (1990). Antimicrobial peptide, tachyplesin I, isolated from hemocytes of the horseshoe crab (*Tachypleus tridentatus*). NMR determination of the beta-sheet structure. J. Biol. Chem..

[36] Nakamura T., Furunaka H., Miyata T., Tokunaga F., Muta T., Iwanaga S., Niwa M., Takao T., Shimonishi Y. (1988). Tachyplesin, a class of antimicrobial peptide from the hemocytes of the horseshoe crab (*Tachypleus tridentatus*). Isolation and chemical structure. J. Biol. Chem..

[37] Miyata T., Tokunaga F., Yoneya T., Yoshikawa K., Iwanaga S., Niwa M., Takao T., Shimonishi Y. (1989). Antimicrobial peptides, isolated from horseshoe crab hemocytes, tachyplesin II, and polyphemusins I and II: Chemical structures and biological activity. J. Biochem..

[38] Mandard N., Sy D., Maufrais C., Bonmatin J.M., Bulet P., Hetru C., Vovelle F. (1999). Androctonin, a novel antimicrobial peptide from scorpion Androctonus australis: Solution structure and molecular dynamics simulations in the presence of a lipid monolayer. J. Biomol. Struct. Dyn..

[39] Kokryakov V.N., Harwig S.S., Panyutich E.A., Shevchenko A.A., Aleshina G.M., Shamova O.V., Korneva H.A., Lehrer R.I. (1993). Protegrins: Leukocyte antimicrobial peptides that combine features of corticostatic defensins and tachyplesins. FEBS Lett..

[40] Deplazes E., Chin Y.K., King G.F., Mancera R.L. (2020). The unusual conformation of cross-strand disulfide bonds is critical to the stability of beta-hairpin peptides. Proteins.

[41] Tanner J.D., Deplazes E., Mancera R.L. (2018). The Biological and Biophysical Properties of the Spider Peptide Gomesin. Molecules.

[42] Troeira Henriques S., Lawrence N., Chaousis S., Ravipati A.S., Cheneval O., Benfield A.H., Elliott A.G., Kavanagh A.M., Cooper M.A., Chan L.Y. (2017). Redesigned Spider Peptide with Improved Antimicrobial and Anticancer Properties. ACS Chem. Biol..

[43] Benfield A.H., Defaus S., Lawrence N., Chaousis S., Condon N., Cheneval O., Huang Y.H., Chan L.Y., Andreu D., Craik D.J. (2021). Cyclic gomesin, a stable redesigned spider peptide able to enter cancer cells. Biochim. Biophys. Acta Biomembr..

[44] Castro J.R., Fuzo C.A., Degreve L., Caliri A. (2008). The role of disulfide bridges in the 3-D structures of the antimicrobial peptides gomesin and protegrin-1: A molecular dynamics study. Genet. Mol. Res..

[45] Rodrigues E.G., Dobroff A.S., Cavarsan C.F., Paschoalin T., Nimrichter L., Mortara R.A., Santos E.L., Fazio M.A., Miranda A., Daffre S. (2008). Effective topical treatment of subcutaneous murine B16F10-Nex2 melanoma by the antimicrobial peptide gomesin. Neoplasia.

[46] Fernandez-Rojo M.A., Deplazes E., Pineda S.S., Brust A., Marth T., Wilhelm P., Martel N., Ramm G.A., Mancera R.L., Alewood P.F. (2018). Gomesin peptides prevent proliferation and lead to the cell death of devil facial tumour disease cells. Cell Death Discov..

[47] Moraes L.G., Fazio M.A., Vieira R.F., Nakaie C.R., Miranda M.T., Schreier S., Daffre S., Miranda A. (2007). Conformational and functional studies of gomesin analogues by CD, EPR and fluorescence spectroscopies. Biochim. Biophys. Acta.

[48] Moreira C.K., Rodrigues F.G., Ghosh A., Varotti Fde P., Miranda A., Daffre S., Jacobs-Lorena M., Moreira L.A. (2007). Effect of the antimicrobial peptide gomesin against different life stages of *Plasmodium* spp.. Exp. Parasitol..

[49] Nadal-Bufi F., Mason J.M., Chan L.Y., Craik D.J., Kaas Q., Troeira Henriques S. (2021). Designed beta-Hairpins Inhibit LDH5 Oligomerization and Enzymatic Activity. J. Med. Chem..

[50] Dias S.A., Pinto S.N., Silva-Herdade A.S., Cheneval O., Craik D.J., Coutinho A., Castanho M., Henriques S.T., Veiga A.S. (2022). A designed cyclic analogue of gomesin has potent activity against *Staphylococcus aureus* biofilms. J. Antimicrob. Chemother..

[51] Domingues T.M., Riske K.A., Miranda A. (2010). Revealing the lytic mechanism of the antimicrobial peptide gomesin by observing giant unilamellar vesicles. Langmuir.

[52] Domingues T.M., Perez K.R., Miranda A., Riske K.A. (2015). Comparative study of the mechanism of action of the antimicrobial peptide gomesin and its linear analogue: The role of the beta-hairpin structure. Biochim. Biophys. Acta.

[53] Freire J.M., Gaspar D., Veiga A.S., Castanho M.A. (2015). Shifting gear in antimicrobial and anticancer peptides biophysical studies: From vesicles to cells. J. Pept. Sci..

[54] Soletti R.C., del Barrio L., Daffre S., Miranda A., Borges H.L., Moura-Neto V., Lopez M.G., Gabilan N.H. (2010). Peptide gomesin triggers cell death through L-type channel calcium influx, MAPK/ERK, PKC and PI3K signaling and generation of reactive oxygen species. Chem. Biol. Interact..

[55] Fazio M.A., Jouvensal L., Vovelle F., Bulet P., Miranda M.T., Daffre S., Miranda A. (2007). Biological and structural characterization of new linear gomesin analogues with improved therapeutic indices. Biopolymers.

[56] Imai K., Takaoka A. (2006). Comparing antibody and small-molecule therapies for cancer. Nat. Rev. Cancer.

[57] Wang L., Wang N., Zhang W., Cheng X., Yan Z., Shao G., Wang X., Wang R., Fu C. (2022). Therapeutic peptides: Current applications and future directions. Signal Transduct. Target Ther..

[58] Smith A.J. (2015). New horizons in therapeutic antibody discovery: Opportunities and challenges versus small-molecule therapeutics. J. Biomol. Screen.

[59] Mahlapuu M., Björn C., Ekblom J. (2020). Antimicrobial peptides as therapeutic agents: Opportunities and challenges. Crit. Rev. Biotechnol..

[60] Elad S., Epstein J.B., Raber-Durlacher J., Donnelly P., Strahilevitz J. (2012). The antimicrobial effect of Iseganan HCl oral solution in patients receiving stomatotoxic chemotherapy: Analysis from a multicenter, double-blind, placebo-controlled, randomized, phase III clinical trial. J. Oral Pathol. Med..

[61] Rader A.F.B., Reichart F., Weinmuller M., Kessler H. (2018). Improving oral bioavailability of cyclic peptides by N-methylation. Bioorg. Med. Chem..

[62] Bech E.M., Pedersen S.L., Jensen K.J. (2018). Chemical Strategies for Half-Life Extension of Biopharmaceuticals: Lipidation and Its Alternatives. ACS Med. Chem. Lett..

